# The effects of coital lubricants on sperm motility and vitality

**DOI:** 10.5935/1518-0557.20240113

**Published:** 2025

**Authors:** Lalita Pradit, Pareeya Somsak, Waraporn Piromlertamorn, Tawiwan Pantasri, Usanee Sanmee

**Affiliations:** 1 Division of Reproductive Medicine, Department of Obstetrics and Gynecology, Faculty of Medicine, Chiang Mai University, Chiang Mai 50200, Thailand; 2 CMEx Fertility Center, Center of Medical Excellence, Chiang Mai University, Chiang Mai 50200, Thailand

**Keywords:** lubricants, spermatozoa, motility, vitality

## Abstract

**Objective:**

To investigate the effects of five coital lubricants including KY jelly, Durex, Pre-seed, Vaseline, and Baby oil on sperm motility and vitality.

**Methods:**

Raw semen samples from 20 normozoospermic donors were incubated in vaginal fluid simulant (VFS) controls and a 10% concentration of KY jelly, Durex, Pre-seed, Vaseline, and Baby oil. The sperm motility and vitality were evaluated immediately after mixing (0 minutes) and at 30 and 60 minutes.

**Results:**

Sperm motility significantly decreased immediately after mixing in all coital lubricants. Pre-seed demonstrated sperm parameters comparable to VFS at any incubation time. KY jelly, Vaseline, and Baby oil reduced the progressive sperm motility at a certain time, both 30 and 60 minutes in KY jelly, at only 30 minutes in Baby oil, and at only 60 minutes in Vaseline. Durex showed a significant decrease in sperm motility at any incubation time and significantly deteriorated sperm vitality at 60 minutes while other lubricants did not affect sperm vitality.

**Conclusions:**

Pre-seed was the coital lubricant that had the least negative effect on sperm. It can be an appropriate coital lubricant for the couple who are trying to conceive when lubricant is indicated. In contrast, KY jelly and Durex are the coital lubricants that should be avoided, especially Durex which had the worst effect on both sperm motility and vitality.

## INTRODUCTION

Dyspareunia or painful sexual intercourse is a common condition in reproductive age women with the prevalence ranging from 8% to 22% (Latthe *et al.*, 2006). The prevalence may be twofold higher in women trying to conceive due to the stress associated with timed intercourse (Ellington *et al.*, 2003). A coital lubricant is an option to overcome this problem by reducing friction in the vagina during intercourse. There are many types of coital lubricants used, including those commercially available on the market, which have varying compositions, pH, and osmolarity, and some claim to not harm sperm, and noncommercial products available at home such as oil used in cooking or nourishing the body. The exposure of semen to coital lubricants is causing concern regarding the possible adverse effect of this lubricant on sperm function affecting its fertilization potential which may decrease the chance of conception.

Many studies found a decrease in sperm motility (Agarwal *et al.*, 2008; Mackenzie & Gellatly, 2019; Sandhu *et al.*, 2014; Soriano *et al.*, 2021), vitality (Agarwal *et al.*, 2008; Mowat *et al.*, 2014), and DNA integrity (Agarwal *et al.*, 2008) after exposure of semen to various coital lubricants even in low concentration for just a few minutes (Miller *et al.*, 1994) while some lubricants reported no deleterious effect on sperm (Agarwal *et al.*, 2008; Markram *et al.*, 2022; Rafaee *et al.*, 2022; Sandhu *et al.*, 2014). This poses a challenge for couples who are trying to conceive but rely on lubricants to choose products that are safe for sperm.

We are interested in the five coital lubricants that are most commonly used in couples trying to conceive according to our survey including KY jelly, Durex, Pre-seed, Vaseline, and Baby oil. We aimed to design the methodology of the study to mimic the in vivo condition when semen is mixed with lubricants. The concentration of the coital lubricants will be diluted by the secretion from the vagina during intercourse before being exposed to the ejaculated semen. Therefore, our in vitro study used the vaginal fluid simulant (VFS) to represent vaginal secretion to adjust the concentration of coital lubricants before exposed to the raw semen samples. Based on our knowledge, this is the first study using VFS as the solvent to prepare coital lubricants for the study. We investigated the effects of these five lubricants on sperm motility and viability because motility is the most important predictor of sperm transport and subsequent fertilization, while vitality determines the toxic effect of lubricant on sperm.

## MATERIALS AND METHODS

### Participants and semen collection

Semen samples were obtained from 20 normozoospermic donors, according to the World Health Organization (WHO, 2021); sperm volume ≥1.4 ml, concentration ≥16x10^6^cells/ml, total sperm motility ≥ 42%, progressive motility ≥30%, normal morphology ≥4%, and leukocyte count <1.0x10^6^cells/ml. Informed consent was obtained before sample collection. Sterile containers for semen collection were given to patients. They collected the semen by masturbation after 2-7 days of abstinence. Routine semen analysis was performed within one hour of production to determine baseline parameters. This study was approved by the Research Ethics Committee of the Faculty of Medicine, Chiang Mai University under approval no. 069/2023.

### Vaginal fluid simulant (VFS)

VFS was developed by Owen & Katz (1999) to have the same physical and chemical properties, particularly pH and osmolarity as native human vaginal fluid. A composition for one liter of VFS contains NaCl 3.51g, KOH 1.40g, Ca(OH)_2_ 0.222g, bovine serum albumin (BSA) 0.018g, lactic acid 2.0g, acetic acid 1.0g, glycerol 0.16g, urea 0.40g, and glucose 5.0g. The mixture is adjusted to a pH of 4.2 and osmolarity of 290 mOsm/L. Once prepared, VFS was stable at 4℃ for one month (Rastogi *et al.*, 2016).

### Lubricant preparation

Five coital lubricants were analyzed: KY jelly (Reckitt Benckiser Healthcare, Italy), Durex (Reckitt Benckiser Healthcare, Thailand), Pre-Seed (Church and Dwight Co., UK), Vaseline (Unilever Thailand), and Baby oil (Johnson and Johnson, Thailand). These coital lubricants were chosen from a survey of the commonly used coital lubricants in couples attending the CMEx Fertility Center, Faculty of Medicine, Chiang Mai University, Thailand. All lubricants were in date at the time of use.

In physiological conditions, the lubricants are potentially present in 5-30% after mixed with vaginal secretion during intercourse (Anderson *et al.*, 1998; Boyers *et al.*, 1987; Miller *et al.*, 1994; Tulandi & McInnes, 1984). Considering the observations of other published studies that mostly used coital lubricants in 10% concentration. Therefore, we designed to use a 10% concentration of coital lubricants in our study, obtained by aspirating 1 mL of the lubricant and subsequently dissolving it with 9 mL of VFS. Each lubricant was mixed thoroughly and then checked the pH and osmolarity. The pH value was determined by a pH meter (GonotecOsmomat 030; Gonotec GmbH, Berlin, Germany). The osmolarity was measured using a digital osmometer (Starter 3100 pH Bench; Ohaus Corporation, New Jersey, USA).

### Sperm motility and vitality assessment

Raw semen was mixed in a 1:1 ratio with each 10% concentration lubricant as the test group and mixed with VFS as the control group. The sperm motility and vitality were evaluated immediately after mixing (0 minutes) and at 30 and 60 minutes. These intervals were selected based on sperm transport physiology that a majority of fertilizing sperm enter cervical mucous within 15 to 30 minutes from ejaculation (Settlage *et al.*, 1973; Suarez & Pacey, 2006). At 60 minutes, almost all the penetrable sperm have left semen (Tomlinson *et al.*, 2023), therefore if toxicity is not observed by this time means it is of no clinical importance.

Sperm motility was assessed using an HTM IVOS II computer-assisted semen analyzer (CASA; Hamilton Thorne Biosciences, Beverly, MA), equipped with Clinical Human Motility II software.

Sperm vitality assessment using eosin staining. Ten µl from each sample was mixed with 10 µl of 0.5% Eosin-Y (Sigma Chemical) on a glass microscopic slide. Eosin stains only the dead sperm, turning them red, whereas the viable sperm appears unstained. At least 200 spermatozoa were counted in duplicates.

### Statistical analysis

Statistical analysis was performed using SPSS program version 27. Data are expressed as mean±standard deviation or median (interquartile range) based on data distribution. The sperm motility and vitality of each lubricant were compared by repeated measures analysis of variance (ANOVA) when data distribution was normal or the Friedman test when normality could not be confirmed. If there was a significant difference, Tukey’s or Dunn’s multiple comparisons test would be done. A *p*-value of <0.05 was considered statistically significant.

## RESULTS

Twenty normozoospermic semen samples were included in this study. The mean age of the participants and baseline sperm parameters are shown in Table 1. The pH and osmolarity in the 10% concentration of each lubricant are presented in Table 2.

**Table 1:** Mean age and sperm parameters.

Parameters	Mean±SD
Age (year)	32.15±5.24
Abstinence (day)	3.90±1.51
Volume (ml)	2.75±0.92
pH	8.30±0.33
Sperm concentration (million/ml)	99.72±55.37
Total motility (%)	70.76±9.81
Progressive motility (%)	63.89±9.53

**Table 2:** pH and osmolarity of lubricant preparation.

Lubricant preparation (10% v/v)	pH	Osmolarity (mOsm/L)
Vaginal fluid simulant	4.58±0.09	294±3.79
KY jelly	4.53±0.07	473±36.86
Durex	4.49±0.05	821±28.48
Pre-seed	4.56±0.08	303±12.50
Vaseline	4.45±0.05	300±8.02
Baby oil	4.51±0.01	305±12.12

Data expressed as mean±standard deviation.

**Figure 1:**
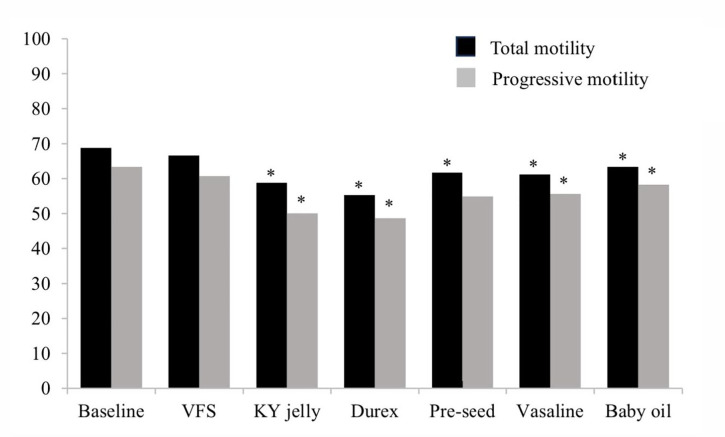
Total and progressive sperm motility immediately (at 0 minute) after expose to each lubricant and control (VFS) compared to baseline sperm parameters. Friedman test, *p<0.05 compared to baseline sperm parameters.

There was no difference in sperm motility after raw semen was exposed to VFS (control) compared to the baseline sperm parameter (Figure 1). However, immediately after exposure of raw semen to lubricant preparation, the sperm motility significantly decreases on both total and progressive motility in KY jelly, Durex, Vaseline, and Baby oil but only significantly decreases in total sperm motility in Pre-seed compared to baseline parameters (Figure 1).

After 30- and 60-minutes incubation, all lubricants and control demonstrated a significant loss of total and progressive sperm motility (*p*<0.001, Table 3). Compared to control, exposure to coital lubricants caused a remarkable decrease in sperm motility, except for Pre-seed, which showed no significant change in total and progressive motility at any time assessed. Durex demonstrated a significant decrease in total and progressive motility in a time dependent manner. KY jelly significantly reduced progressive motility compared to control at any time assessed. Vaseline significantly decreases progressive motility at 60 minutes. Baby oil significantly decreases total and progressive motility at 30 minutes after incubation (Table 3).

**Table 3:** Total and progressive sperm motility at 0, 30, and 60 minutes after incubation with each lubricant and control (VFS).

Time (minute)	Lubricants						p-value
	VFS	KY jelly	Durex	Pre-Seed	Vaseline	Baby oil	
**Total sperm motility**							
0	66.58 (49.47-75.22) ^*^	58.84 (34.81-69.85)	55.33 (33.16-63.33) ^*^	61.68 (39.58-69.91)	61.20 (31.93-70.75)	63.42 (36.95-66.61)	0.001
30	38.18 (17.22-52.80) ^†,‡^	24.47 (16.55-43.36)	17.06 (8.26-34.75) ^†^	22.99 (16.25-42.61)	27.25 (13.59-36.51)	23.64 (11.45-46.91) ^‡^	0.009
60	17.79 (3.05-27.37) ^§^	11.18 (1.27-20.43)	5.57 (2.55-22.43) ^§^	14.66 (2.24-30.11)	12.00 (0.13-30.93)	10.26 (2.67-21.36)	0.008
**p-value**	<0.001	<0.001	<0.001	<0.001	<0.001	<0.001	
**Progressive sperm motility**							
0	60.71 (42.62-68.44)	50.06 (26.38-64.19)	48.65 (30.80-58.52)	54.86 (31.67-63.39)	55.60 (23.28-66.62)	58.34 (28.53-63.33)	<0.001
30	32.18 (13.16-48.38) ^‖,¶,**^	19.09 (9.73-32.37) ^‖^	11.72 (6.32-28.60) ^¶^	17.88 (11.94-37.70)	20.73 (9.41-31.15)	16.95 (7.84-42.10) ^**^	0.001
60	12.88 (1.57-23.76) ^††,§§,‖‖^	5.50 (0.43-12.46) ^††^	3.93 (1.84-17.86) ^§§^	10.98 (1.57-20.52)	7.03 (0.00-21.71) ^‖‖^	5.89 (1.79-16.70)	0.001
**p-value**	<0.001	<0.001	<0.001	<0.001	<0.001	<0.001	

Data expressed as median (interquartile range)

Friedman test,

* ,^‖^*p*=0.015,

† *p*=0.006,

‡,†† *p*=0.046,

§ *p*=0.041,

¶ *p*=0.002,

** *p*=0.035,

§§ *p*<0.001,

‖‖ *p*=0.020.

Regarding sperm vitality, all lubricants and control demonstrated a significant loss of sperm vitality at 60 minutes (*p*<0.001, Table 4). However, a significant reduction in sperm vitality at 30 minutes was found only in Durex. Furthermore, Durex showed significantly lower sperm vitality at 60 minutes compared to the control. In contrast, sperm vitality after incubating with other lubricants was comparable to control at any time assessed (Table 4).

**Table 4:** Sperm vitality at 0, 30, and 60 minutes after incubation with each lubricant and control (VFS).

Time (minute)	Lubricants						p-value
	VFS	KY jelly	Durex	Pre-Seed	Vaseline	Baby oil	
0	96.50 (92.75-98.00)	95.00 (88.25-96.75)	94.00 (89.00-97.00)	96.50 (89.25-98.00)	95.50 (88.00-96.75)	92.50 (91.25-94.75)	0.281
30	92.00 (86.25-94.75)	91.50 (85.00-94.75)	90.05 (86.00-94.00)	95.00 (87.00-96.75)	92.50 (88.25-95.75)	92.00 (87.25-94.75)	0.060
60	90.05 (85.75-94.00) ^*^	88.00 (74.25-93.00)	83.00 (64.25-90.05) ^^*^,†^	91.50 (85.25-93.75) ^†^	87.00 (81.00-91.50)	87.50 (76.50-92.00)	0.001
***p*-value**	0.007	0.004	<0.001	0.001	0.006	0.016	

Data expressed as median (interquartile range)

Friedman test,

* *p*=0.005,

† *p*=0.008.

## DISCUSSION

We searched the published study on the five coital lubricants most commonly used in our survey from couples who are trying to conceive: KY jelly, Durex, Pre-seed, Vaseline, and Baby oil. There are several studies consistently showing the negative effect of KY jelly on sperm motility (Goldenberg & White, 1975; Mackenzie & Gellatly, 2019; Sandhu *et al.*, 2014; Soriano *et al.*, 2021; Tomlinson *et al.*, 2023) and vitality (Agarwal *et al.*, 2008). Studies demonstrated the detrimental effects of Durex (Tomlinson *et al.*, 2023), Vaseline (Goldenberg & White, 1975), and Baby oil (Sandhu *et al.*, 2014) on sperm motility are limited and there is no data on sperm vitality. Inconsistent data was found in Pre-seed which is considered as sperm friendly lubricant. Some studies showed no effect on sperm motility (Agarwal *et al.*, 2008; Mowat *et al.*, 2014) and vitality (Agarwal *et al.*, 2008), but others showed the reduction of sperm motility with Pre-seed (Markram *et al.*, 2022; Sandhu *et al.*, 2014). The variations in study methodology make quantitative review unsuitable such as [1] variety in the solvent used to prepare lubricant concentrations: sperm washing media (Mackenzie & Gellatly, 2019; Tomlinson *et al.*, 2023), human tubal fluid (Agarwal *et al.*, 2008; Sandhu *et al.*, 2014), in vitro fertilization (IVF) media (Soriano *et al.*, 2021) or normal saline (Goldenberg & White, 1975), [2] variety in sperm used in the study: raw sperm (Agarwal *et al.*, 2008; Goldenberg & White, 1975; Markram *et al.*, 2022) or prepared sperm with density gradient centrifugation (Mackenzie & Gellatly, 2019; Sandhu *et al.*, 2014; Tomlinson *et al.*, 2023) or swim up (Soriano *et al.*, 2021), and [3] variety in time to assessed sperm function. Therefore, the present project aimed to analyze the effect of coital lubricant under conditions that mimic normal physiology that lubricant be diluted by vaginal secretion before exposure to raw (untreated) sperm. To our knowledge, this is the first study that used VFS with similar properties, compositions and concentrations of constituents found in human vaginal fluid.

The result of this study showed a significant reduction in sperm motility immediately after sperm were exposed to all types of coital lubricant, only VFS did not have a harmful on sperm motility. Therefore, the present finding suggested avoidance of all types of coital lubricant is the best approach in couples attempting to conceive. However, if coital lubricant is indicated, Pre-seed had the least effect compared to other lubricants. When incubation time passed, Pre-seed demonstrated sperm parameters comparable to VFS at any incubation time representing that it had the least negative effect on sperm. KY jelly, Vaseline, and Baby oil reduced the progressive sperm motility at a certain time, both 30 and 60 minutes in KY jelly, at only 30 minutes in Baby oil, and at only 60 minutes in Vaseline. Durex showed a significant decrease in sperm motility at any incubation time. Moreover, only Durex had deteriorated sperm vitality, while other lubricants did not affect sperm vitality. However, Soriano *et al.* (2021) achieved contradictory results showing that Durex initiates hyperactive motility the entire incubation time and suggests being a lubricant of choice for infertile couples.

A possible explanation for sperm damage after exposed to coital lubricants is most likely due to the nonphysiologic osmolarity of the lubricants. Even diluted with vaginal secretions, the osmolarity remains high in Durex and KY jelly (821±28 and 473±36 mOsm/L, respectively) which is compatible with the results of the study that the worst sperm parameter was observed in Durex, and secondly in KY jelly. The optimal osmolarity for sperm function is between 270 and 360 mOsm/L (Agarwal *et al.*, 2008). Outside this range will harm sperm motility. Rossato *et al.* (2002) demonstrated a significant linear negative correlation between semen osmolarity and sperm motility. They found that sperm incubation in media with high osmolarity leads to a progressive decrease in sperm motility, a 50% reduction when the osmolarity was above 400mOsm/L, and almost completely immotile when osmolarity was higher than 600mOsm/L. Therefore, an iso-osmolar lubricant is a good option to avoid such negative effects.

Another possible cause of sperm damage is the toxic components of lubricants. Glycerin is the main ingredient found in most water-based lubricants including Durex and KY jelly which can penetrate across sperm membranes, dissolve the sperm tails flagellar membrane, and disrupt sperm motility (Boyers *et al.*, 1987; Gilmore *et al.*, 1997; Tulandi & McInnes, 1984; Vargas *et al.*, 2011; Wilson *et al.*, 2017). In addition to the fact that glycerin typically causes hypertonicity and contributes to an increase in the osmolarity of the product, it also has a direct toxic effect on sperm. Pre-seed which does not contain glycerin was not toxic. Therefore, couples who trying to conceive should avoid glycerin containing coital lubricant. Our study found that oil-based coital lubricants also harm sperm motility. This might be due to the direct toxicity of oil itself to sperm. Vaseline has less effect on sperm motility than Baby oil because it decreases sperm motility at 60 minutes unlike at 30 minutes in Baby oil, at this time point would not physiologically affect fertility (Overstreet *et al.*, 1980). There are many commercial lubricants available in the market with various components that may have different effects on sperm function. The study of specific components in lubricant rather than glycerin on sperm function is still lacking and remains an ongoing area of research.

The change in normal pH values, ranging from 7.2 to 8.5, can also affect sperm motility. A significant reduction in sperm motility is seen at a pH level less than 6.0 (Makler *et al.*, 1981; Peek & Matthews, 1986; Zavos & Cohen, 1980). Unfortunately, a healthy human vagina is acidic with a pH ranging from 3.2 to 4.2 providing protection against pathogenic microbials. Therefore, the normal environment of the vagina is not suitable for the survival of sperm. Consistent with our study found that sperm motility decreases over time even in the VFS group which is probably from an acidic pH condition. There is still debate about the suitable pH range for coital lubricant (Mowat *et al.*, 2014) which is not only safe for sperm but also does no harm to the vaginal flora. However, even though making the product to a neutral nor basic pH which suitable for sperm, it also becomes acidic when mixed with vaginal secretion which increases during intercourse. Moreover, there is a natural self-protection mechanism in normal physiology that the ejaculated semen coagulates within a minute and forms gel to protect sperm against the harsh vaginal environment including acidity. The coagulation is then enzymatic digested gradually to let the sperm leave the seminal fluid and enter the cervical canal within a few minutes of vaginal deposition (Overstreet *et al.*, 1980). Furthermore, the effect of acidic pH on sperm function is almost completely reversible when pH is restored to the basic pH level in the cervical canal (Makler *et al.*, 1981). Therefore, the pH of the coital lubricant seems to be less valuable than osmolarity.

In this study, we found that Pre-seed had the least negative effect on sperm motility and vitality due to the presence of nontoxic ingredients with the iso-osmolar property. The second least harmful effect is an oil-based lubricant, with Vaseline being more preferred than Baby oil. In contrast, KY jelly and Durex have significant negative effects on sperm, especially Durex is the most detrimental lubricant to sperm, not only on sperm motility but also affect vitality.

Sperm detrimental effect does not only depend on the type of lubricant and time exposed but also depends on the concentration of lubricant (Miller *et al.*, 1994). Our study only tests the result of 10% concentration and suggests that Pre-seed is the coital lubricant that is harmless for sperm, but the effect on sperm at higher concentrations cannot be determined. The intravaginal lubricant concentration achieved varies depending on the vaginal secretion volume among women. Therefore, we recommended using coital lubricant in as little amount as possible only when necessary for adequate coitus in a couple attempting to conceive.

In conclusion, Pre-seed was the coital lubricant that had the least negative effect on sperm. It can be an appropriate coital lubricant for the couple who are trying to conceive when lubricant is indicated. In contrast, KY jelly and Durex are the coital lubricants that should be avoided, especially Durex which had the worst effect on both sperm motility and vitality.
